# Adrenergic supersensitivity and impaired neural control of cardiac electrophysiology following regional cardiac sympathetic nerve loss

**DOI:** 10.1038/s41598-020-75903-y

**Published:** 2020-11-02

**Authors:** Srinivas Tapa, Lianguo Wang, Samantha D. Francis Stuart, Zhen Wang, Yanyan Jiang, Beth A. Habecker, Crystal M. Ripplinger

**Affiliations:** 1 Department of Pharmacology, UC Davis School of Medicine, 2419B Tupper Hall, One Shields Ave, Davis, CA 95616 USA; 2 Department of Chemical Physiology and Biochemistry, Oregon Health and Science University, Portland, OR USA

**Keywords:** Arrhythmias, Ventricular fibrillation, Ventricular tachycardia, Neurodegeneration

## Abstract

Myocardial infarction (MI) can result in sympathetic nerve loss in the infarct region. However, the contribution of hypo-innervation to electrophysiological remodeling, independent from MI-induced ischemia and fibrosis, has not been comprehensively investigated. We present a novel mouse model of regional cardiac sympathetic hypo-innervation utilizing a targeted-toxin (dopamine beta-hydroxylase antibody conjugated to saporin, DBH-Sap), and measure resulting electrophysiological and Ca^2+^ handling dynamics. Five days post-surgery, sympathetic nerve density was reduced in the anterior left ventricular epicardium of DBH-Sap hearts compared to control. In Langendorff-perfused hearts, there were no differences in mean action potential duration (APD_80_) between groups; however, isoproterenol (ISO) significantly shortened APD_80_ in DBH-Sap but not control hearts, resulting in a significant increase in APD_80_ dispersion in the DBH-Sap group. ISO also produced spontaneous diastolic Ca^2+^ elevation in DBH-Sap but not control hearts. In innervated hearts, sympathetic nerve stimulation (SNS) increased heart rate to a lesser degree in DBH-Sap hearts compared to control. Additionally, SNS produced APD_80_ prolongation in the apex of control but not DBH-Sap hearts. These results suggest that hypo-innervated hearts have regional super-sensitivity to circulating adrenergic stimulation (ISO), while having blunted responses to SNS, providing important insight into the mechanisms of arrhythmogenesis following sympathetic nerve loss.

## Introduction

Myocardial infarction (MI) is the leading cause of death globally for both males and females. Though improvements in medical therapies have increased survival rates after MI, approximately 40% of patients will experience post-MI complications, including ventricular arrhythmias^1–4^. Arrhythmias arise due to cardiomyocyte death and subsequent electrophysiological and fibrotic remodeling, increasing the risk for irregular activation and repolarization^5–8^. Moreover, the sympathetic nervous system (SNS) also undergoes dramatic post-MI remodeling, which leads to altered excitability, density, distribution, and neurotransmitter content of cardiac sympathetic fibers^7,9–11^. This interplay between post-MI electrophysiological and sympathetic remodeling is not well understood.

Clinical evidence in post-MI patients suggests that the extent of hypo-innervated surviving myocardium is a better predictor of ventricular arrhythmias and sudden cardiac death (SCD) than infarct size or ejection fraction^12–16^. Our previous experimental work in the mouse heart demonstrated that the infarct region remains devoid of sympathetic fibers due to the presence of chondroitin sulfate proteoglycans (CSPGs), which inhibit reinnervation^11,17^. We further showed that the hypo-innervated infarct region has supra-physiological β-adrenergic receptor (β-AR) responses, including dramatic action potential duration (APD) shortening, diastolic Ca^2+^ elevation, and premature ventricular complexes (PVCs) in response to circulating β-AR agonists. Importantly, this arrhythmogenic phenotype was almost completely reversed when sympathetic re-innervation of the infarct was produced with either genetic deletion or pharmacologic inhibition of the neuronal receptor for CSPGs, the protein tyrosine phosphatase receptor σ (PTPRσ)^11^.

These data suggest an important and fundamental role for sympathetic nerve loss in post-MI arrhythmogenesis, which may be due to adrenergic super-sensitivity and subsequent supra-physiological responses of the surviving myocardium. However, because these hearts had MI and accompanying nerve loss, it was not possible to precisely assess the impact of sympathetic hypo-innervation independent of ischemia-induced electrophysiological and fibrotic remodeling. Therefore, the goal of this study was to directly investigate the role of regional sympathetic hypo-innervation on ventricular electrophysiology (EP) and Ca^2+^ handling in the absence of infarction.

Several experimental approaches have been used previously to study the cardiovascular effects of sympathetic nerve loss. Most commonly, systemic administration of reserpine or 6-hydroxydopamine (6-OHDA) has been used to deplete cardiac norepinephrine (NE) or destroy catecholaminergic neurons, respectively^18,19^. Reserpine, however, can also deplete catecholamines in the central nervous system^20^, which may lead to behavioral effects, and systemic administration of either of these compounds does not produce the regional ventricular nerve loss observed following MI. To achieve regional sympathetic nerve loss, direct application of phenol to the heart has been studied^21^, but this method is not specific to sympathetic neurons and can also damage sensory and parasympathetic neurons as well as cardiomyocytes. Therefore, we developed a novel technique using targeted sympathetic lesioning with an anti-dopamine beta-hydroxylase antibody conjugated to the toxin saporin (anti-DBH-Sap), followed by whole-heart and innervated heart optical mapping to assess electrophysiological dynamics and arrhythmogenesis.

## Results

### Anti-DBH-Sap targets cardiac sympathetic nerve fibers and causes regional hypo-innervation

**Figure 1:**
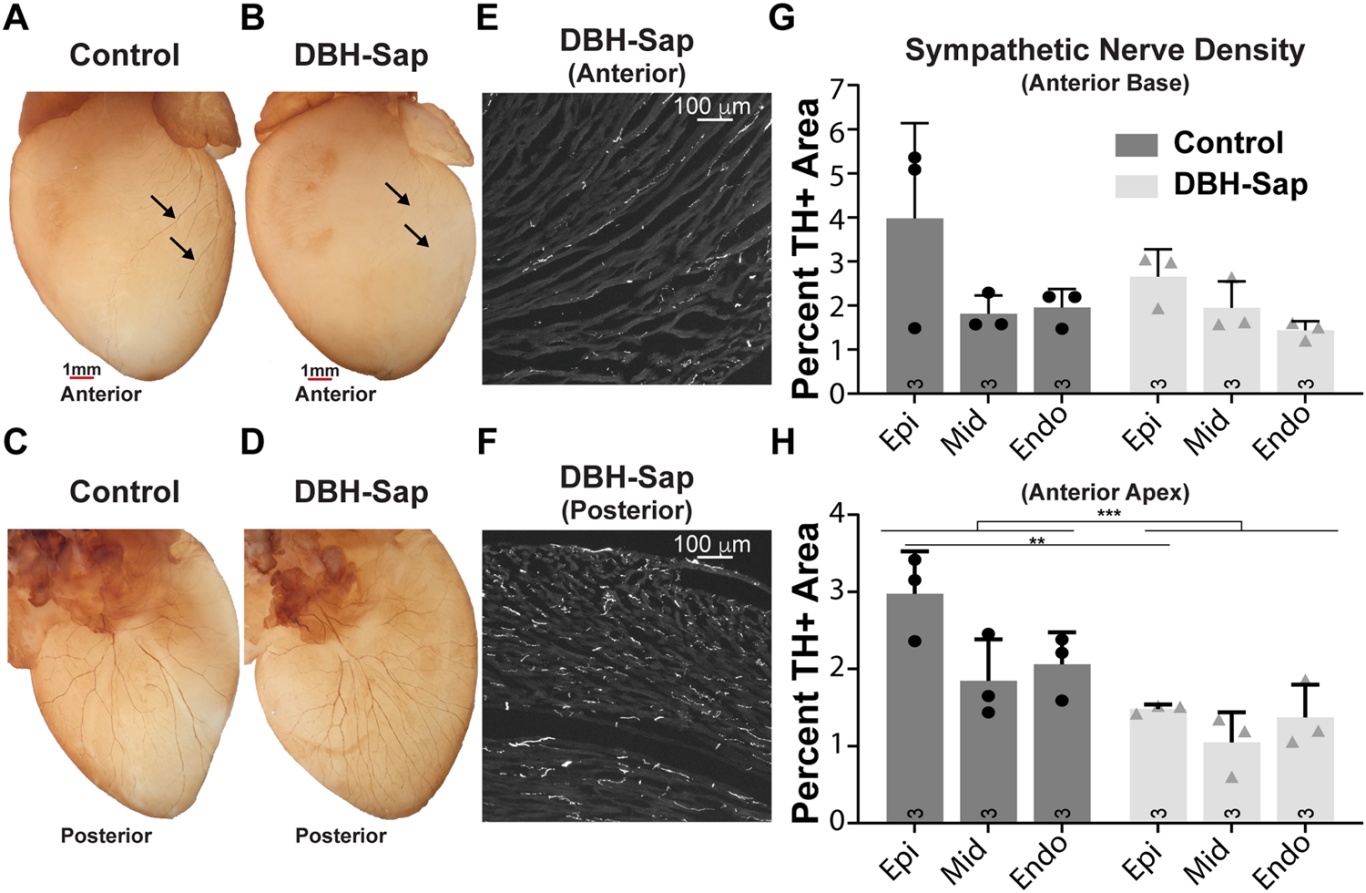
Effects of regional toxin application on sympathetic nerve fiber density. (**A**,**B**) Whole-heart labeling of tyrosine hydroxylase (TH) demonstrates fewer visible sympathetic nerve fibers on the anterior surface (where toxin solution is applied) of the DBH-Sap heart (**B**) compared to control (**A**). (**C**,**D**) The posterior surface is similar between control and DBH-Sap groups. (**E**,**F**) Short axis TH images of the anterior (**E**) and posterior (**F**) surface of a DBH-Sap mouse heart. (**G**,**H**) Sympathetic nerve fiber density from anterior base (**G**) and apex (**H**) regions 5 days after treatment. At the apex, the percent TH + area was significantly reduced in the epicardium of the DBH-Sap group, and there was also a main effect between groups (***p < 0.001, main effect control vs. DBH-Sap, two-way ANOVA, **H**). Data are mean ± SD; analyzed with GraphPad Prism 8.3 (GraphPad Software, San Diego, CA, USA); control: n = 3; DBH-Sap: n = 3; **p < 0.01, ***p < 0.001.

**Figure 2:**
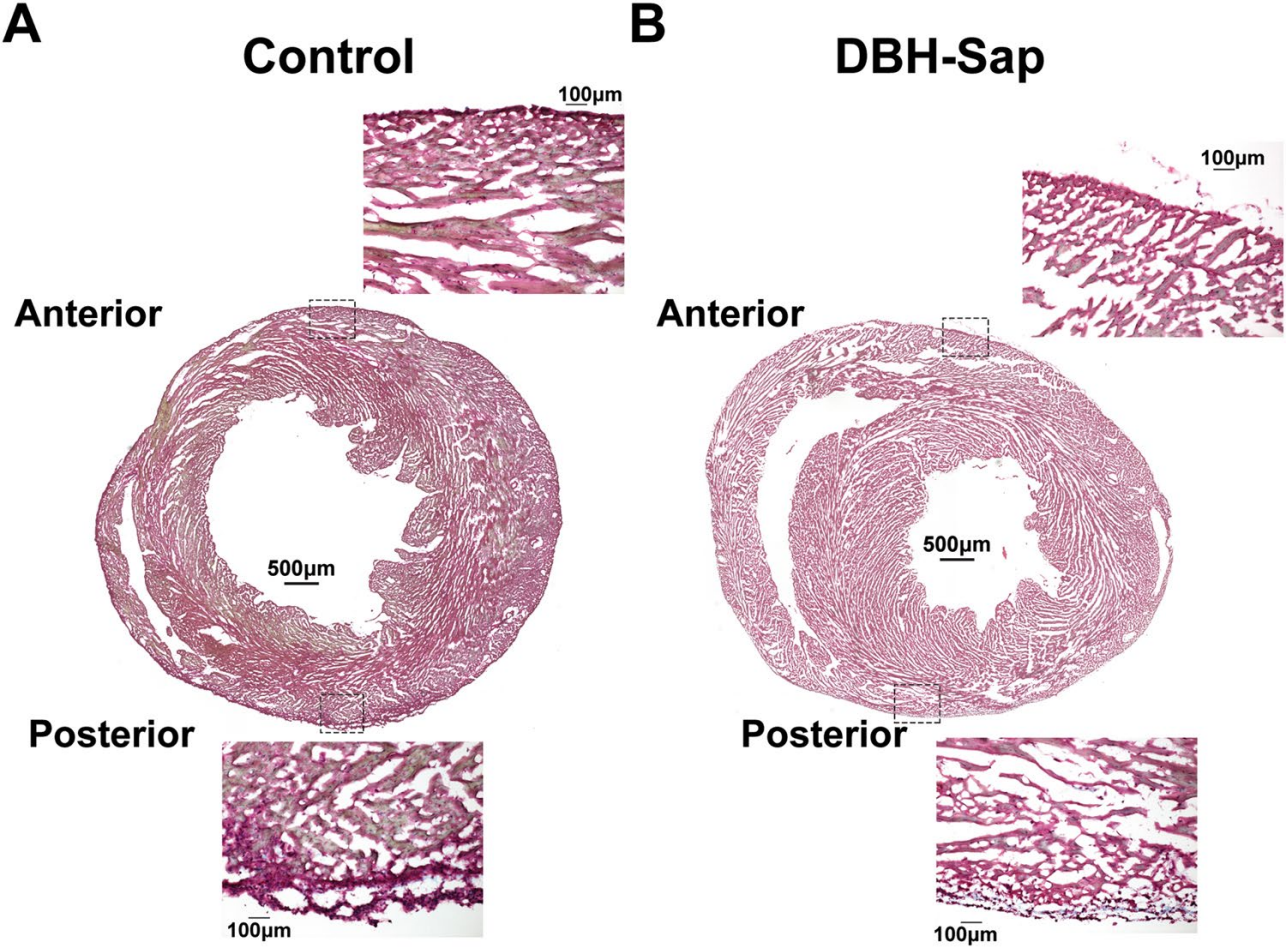
Masson’s trichrome staining of short-axis sections. (**A**) Cross-sectional staining of a control heart and 20 × images of the epicardial/sub-epicardial regions from anterior and posterior surfaces of the heart. (**B**) Cross-sectional staining of a DBH-Sap (toxin-exposed) heart and 20 × images of the epicardial/sub-epicardial regions from anterior and posterior surfaces. No overt myocardial damage was observed in any heart from either group.

Anti-DBH-Sap has been used previously to lesion noradrenergic nerves in the brain and periphery to alter sympathetic control of peripheral organs including the heart, lung, and kidney^22–26^; however, to our knowledge it has never been used to target the intrinsic cardiac sympathetic nerves. Whole-heart labeling of tyrosine hydroxylase (TH) demonstrated visible sympathetic nerve loss on the anterior apical surface of the left ventricle (where anti-DBH-Sap was applied) of toxin-exposed compared to control hearts (Fig. 1A,B), while the posterior regions were not impacted (Fig. 1C,D). Sympathetic nerve density (TH + area) was quantified from short-axis frozen sections (Fig. 1E,F) and a significant reduction (approximately 50%) in the total TH + tissue area was observed at the epicardium of the anterior apex of the DBH-Sap group compared to control (Fig. 1H), while the basal anterior regions show no differences between groups (Fig. 1G). Masson’s trichrome staining demonstrated no overt tissue damage in either group (Fig. 2A,B).

### Regional hypo-innervation causes heterogenous responses to β-AR stimulation with ISO

**Figure 3:**
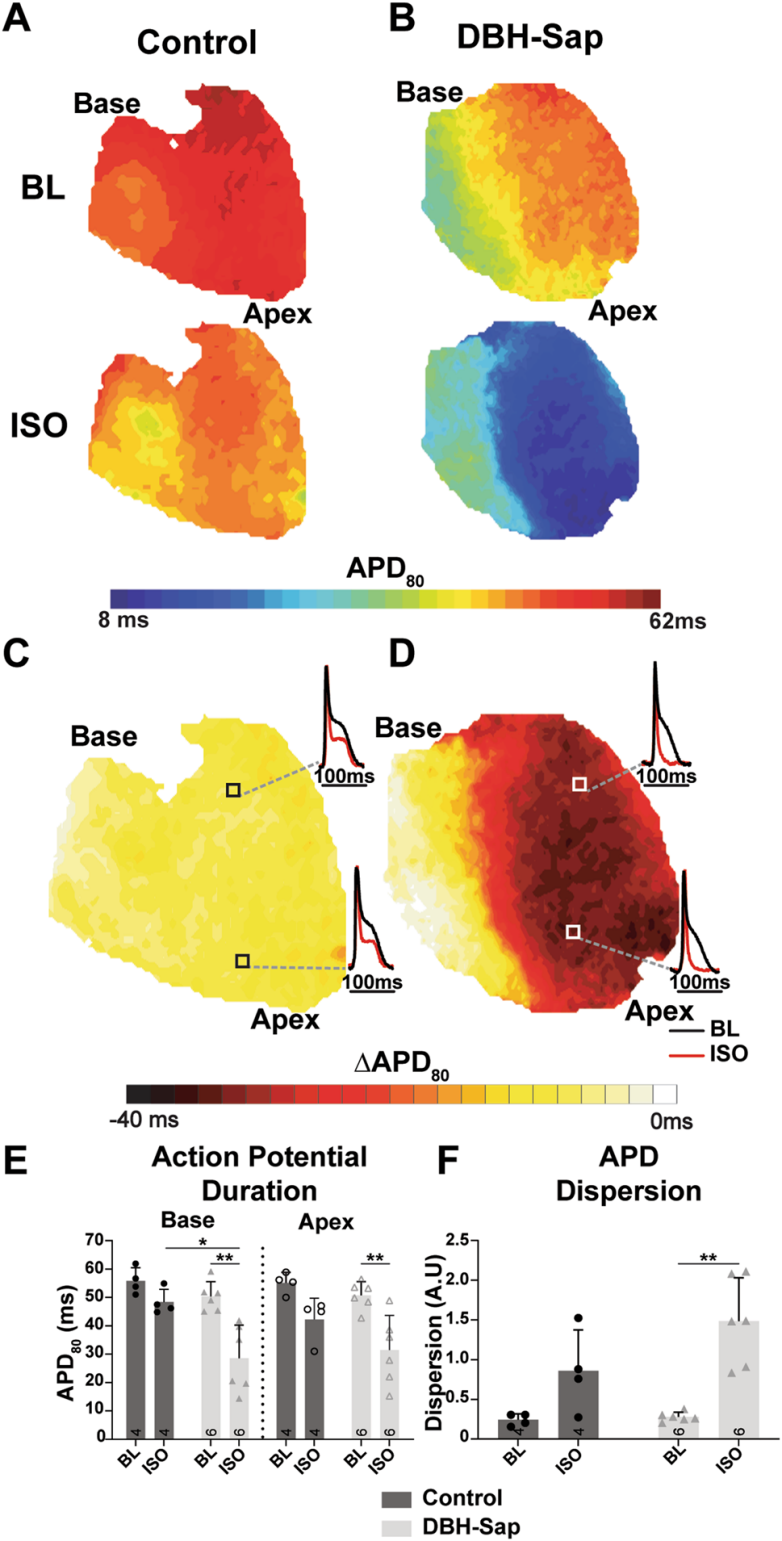
Impact of isoproterenol (ISO) stimulation on action potential duration (APD_80_) and APD dispersion at a pacing cycle length of 100 ms. (**A**,**B**) Example maps of APD_80_ at baseline (BL) and with ISO in both groups. (**C**,**D**) Example maps and optical AP signals showing the difference in APD (ΔAPD_80_) from BL with ISO stimulation. (**E**) Mean APD_80_ at BL and with ISO from base and apex regions of both groups. (**F**) Mean APD dispersion at BL and with ISO in both groups. Data are mean ± SD; analyzed with GraphPad Prism 8.3 (GraphPad Software, San Diego, CA, USA); control: n = 4; DBH-Sap: n = 6; **p < 0.01.

Electrophysiological and Ca^2+^ handling responses to β-AR stimulation were first assessed in traditional Langendorff-perfused hearts with optical mapping and isoproterenol perfusion (ISO: 300 nm–1 µm, Fig. 3). APDs were not different at baseline between the control and DBH-Sap hearts, and no apico-basal differences were noted (baseline APD_80_: control base: 55.8 ± 4.6 ms; control apex: 55.1 ± 3.7 ms; DBH-Sap base: 50.3 ± 5.2 ms; DBH-Sap apex: 50.7 ± 4.8 ms, p = NS between groups and locations). ISO perfusion led to modest and relatively uniform APD shortening in control hearts (Fig. 3A,C,E, ISO APD_80_: control base: 48.3 ± 4.5 ms; control apex: 42.2 ± 7.5 ms, p = NS vs. control baseline). DBH-Sap hearts, on the other hand, showed more dramatic differences in APD responses to ISO, with significant shortening in the hypo-innervated left ventricle (LV, Fig. 3B,D,E, ISO APD_80_: DBH-Sap base: 28.5 ± 11.7 ms, p < 0.01 vs. DBH-Sap baseline and p < 0.05 vs. control ISO; DBH-Sap apex: 31.4 ± 12.2 ms, p < 0.01 vs. DBH-Sap baseline). These data suggest that regions of nerve loss may be more sensitive to β-AR stimulation (Fig. 3E). This differential response to ISO also led to a significant increase in dispersion of APD in the DBH-Sap group but not control (Fig. 3F).

**Figure 4:**
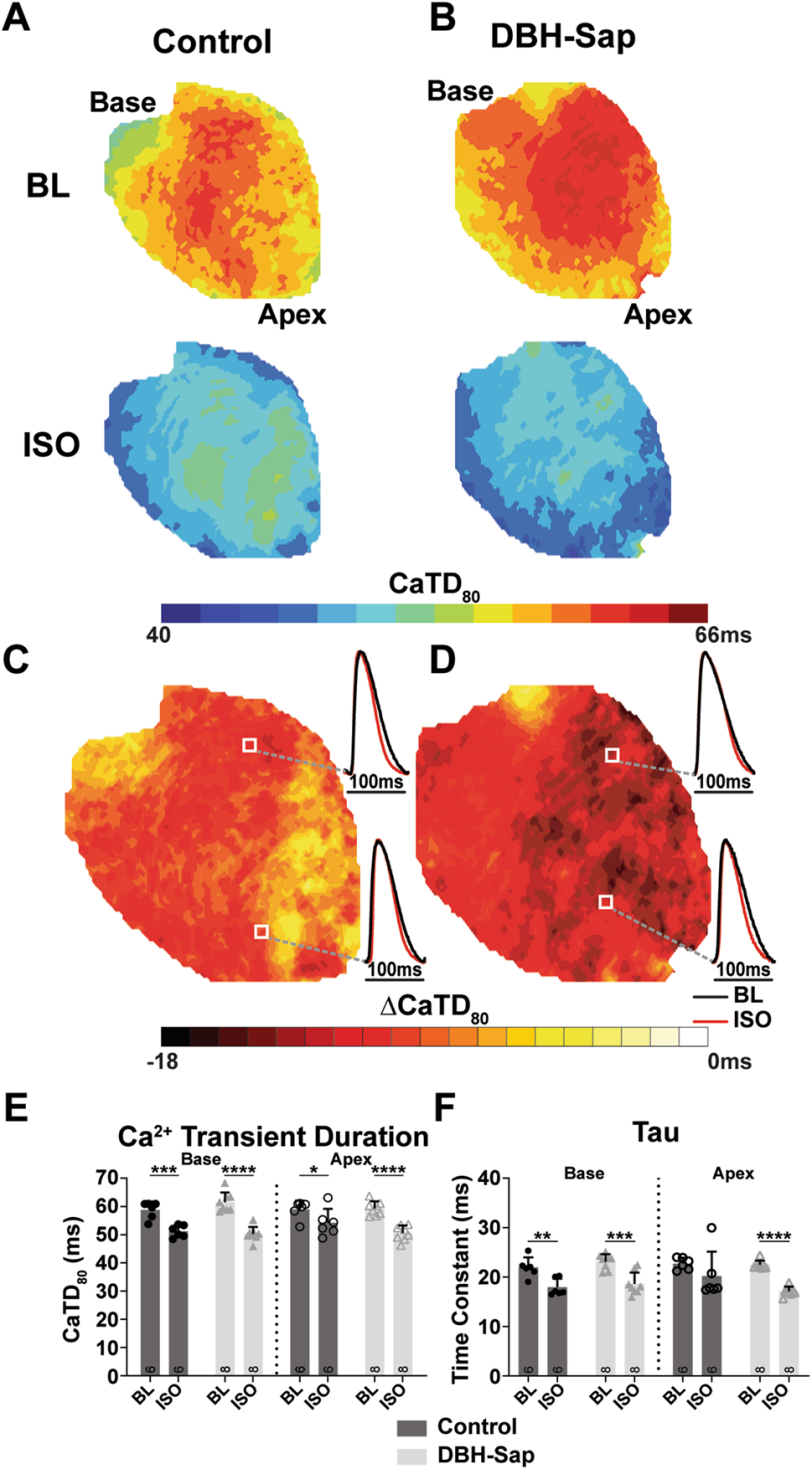
Calcium transient duration (CaTD_80_) and *tau* in control and DBH-Sap hearts at baseline (BL) and with isoproterenol (ISO) stimulation at a pacing cycle length of 100 ms. (**A**,**B**) Example maps of CaTD_80_ at BL and with ISO in both groups. (**C**,**D**) Example maps and optical Ca^2+^ signals showing the difference in CaTD (ΔCaTD_80_) from BL with ISO stimulation. (**E**) Mean CaTD_80_ at BL and with ISO from base and apex regions of both groups. (**F**) Mean *tau* at BL and with ISO for both groups. Data are mean ± SD; analyzed with GraphPad Prism 8.3 (GraphPad Software, San Diego, CA, USA); control: n = 6; DBH-Sap: n = 8; *p < 0.05, **p < 0.01, ***p < 0.001, ****p < 0.0001.

Both the Ca^2+^ transient duration (CaTD) and the time constant of CaT decay (*tau*) are indicators of SERCA pump activity, with shorter CaTD and faster *tau* typically associated with increased SERCA activity. There were no differences in baseline CaTD in control and DBH-Sap hearts and no apico-basal differences were noted (Fig. 4A,B, baseline CaTD_80_: control base: 58.7 ± 3.0 ms; control apex: 58.9 ± 3.2 ms; DBH-Sap base: 61.5 ± 3.4 ms; DBH-Sap apex: 59.3 ± 2.5 ms, p = NS between groups and locations). While the APD maps showed heterogenous regional shortening in response to ISO, the CaTD shortened similarly in both control and DBH-Sap hearts (Fig. 4A–E, ISO CaTD_80_: control base: 51.2 ± 2.2 ms, p < 0.001 vs. baseline; control apex: 54.2 ± 4.9 ms, p < 0.05 vs. baseline; DBH-Sap base: 50.2 ± 2.5 ms, p < 0.0001 vs. baseline; DBH-Sap apex: 50.6 ± 2.6 ms, p < 0.0001 vs. baseline). Likewise, *tau* also shortened similarly in response to ISO in the base of both groups (Fig. 4F, left), but not the apex (Fig. 4F, right).

### β-AR stimulation causes supraphysiologic diastolic Ca^2+^ elevation in hypo-innervated regions

**Figure 5:**
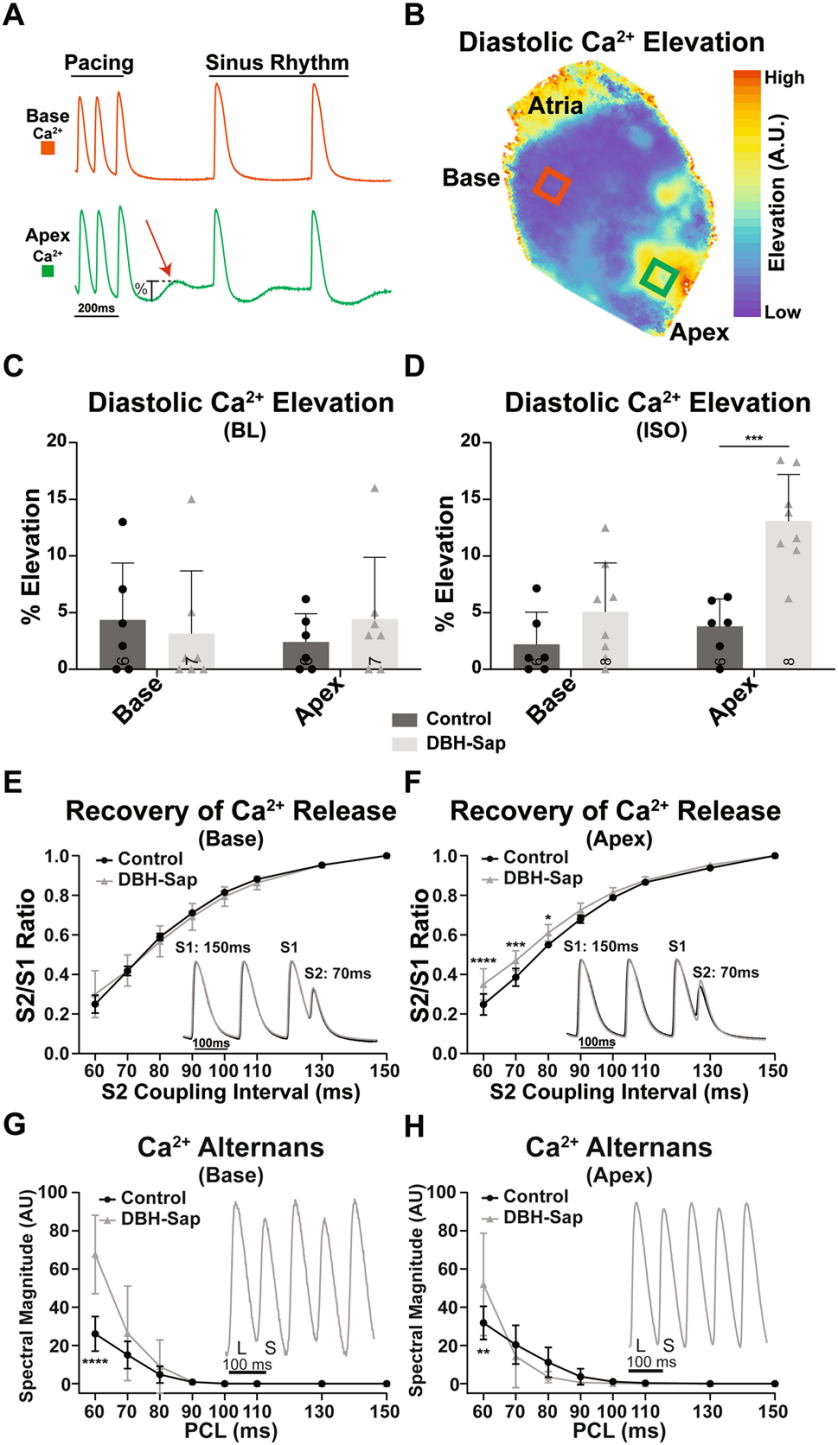
(**A**) Example optical Ca^2+^ signals showing diastolic Ca^2+^ elevation primarily at the apex but not at the base of a DBH-Sap heart following cessation of rapid pacing (70 ms pacing cycle length, PCL). (**B**) Example optical map of relative diastolic Ca^2+^ elevation in a DBH-Sap heart immediately after cessation of pacing at a PCL of 70 ms. (**C**,**D**) Mean diastolic Ca^2+^ elevation at BL (**C**) and with ISO (**D**) from the base and apex regions of both group after a 70 ms pacing train. (**E**) Amplitude of recovery of Ca^2+^ release at the base with progressively shorter S2 coupling intervals. (**F**) Amplitude of recovery of Ca^2+^ release at the apex with progressively shorter S2 coupling intervals. There was a main effect of animal group (p < 0.0001 control vs. DBH-Sap) and differences at S2 = 60, 70, and 80 ms as indicated. (**G**) CaT alternans magnitude (*L* large; *S* small) between control and DBH-Sap hearts at the base. There was a main effect of animal group (p = 0.0002 control vs. DBH-Sap) and a difference at PCL = 60 ms as indicated. (**H**) CaT alternans magnitude at the apex. There was no main effect of animal group, but alternans magnitude was different at PCL = 60 ms. Data in (**E**–**H**) were analyzed by a two-way ANOVA (S2 coupling interval or PCL as one factor, animal group as the other factor) followed by Sidak’s multiple comparison post-testing. Data are mean ± SD; analyzed with GraphPad Prism 8.3 (GraphPad Software, San Diego, CA, USA); control: n = 5–6; DBH-Sap: n = 4–8; *p < 0.05, **p < 0.01, ***p < 0.001, ****p < 0.0001.

Ca^2+^ dynamics were also assessed with rapid pacing (pacing cycle length [PCL] = 70 ms) followed by a pause at baseline and with ISO perfusion (Fig. 5A). An example DBH-Sap heart shows elevation in diastolic Ca^2+^ at the apex compared to basal region following ISO perfusion (Fig. 5B). As expected, diastolic Ca^2+^ elevation was modest and there were no significant differences between groups at baseline (Fig. 5C). Following ISO, however, the apex region of the DBH-Sap group had significantly increased diastolic Ca^2+^ elevation compared to control hearts (Fig. 5D; apex DBH-Sap: 13.08 ± 4.11%; control: 3.78 ± 2.43%*,* p =  < 0.0002).

### Recovery of Ca^2+^ release is accelerated with hypo-innervation, while severity of Ca^2+^ alternans worsens at rapid rates

The relative recovery of Ca^2+^ release was assessed with premature stimuli. At the base, the recovery of Ca^2+^ release was identical between the two groups (Fig. 5E); while at the apex, the relative Ca^2+^ release recovered faster in DBH-Sap hearts (S2/S1 ratio was larger at short coupling intervals), potentially suggesting accelerated recovery of ryanodine receptors (RyR) from refractoriness (Fig. 5F). We previously demonstrated that accelerated recovery of Ca^2+^ release is associated with decreased propensity for arrhythmogenic Ca^2+^ alternans^27^; thus, we hypothesized that the hypo-innervated apical region may have reduced alternans. Contrary to this hypothesis, both groups had similar magnitude alternans up until the fastest PCL (60 ms), in which the DBH-Sap hearts had significantly increased alternans magnitude at both the apex and base (Fig. 5G,H).

### Regional hypo-innervation blunts electrophysiological responses to physiological sympathetic nerve stimulation

**Figure 6:**
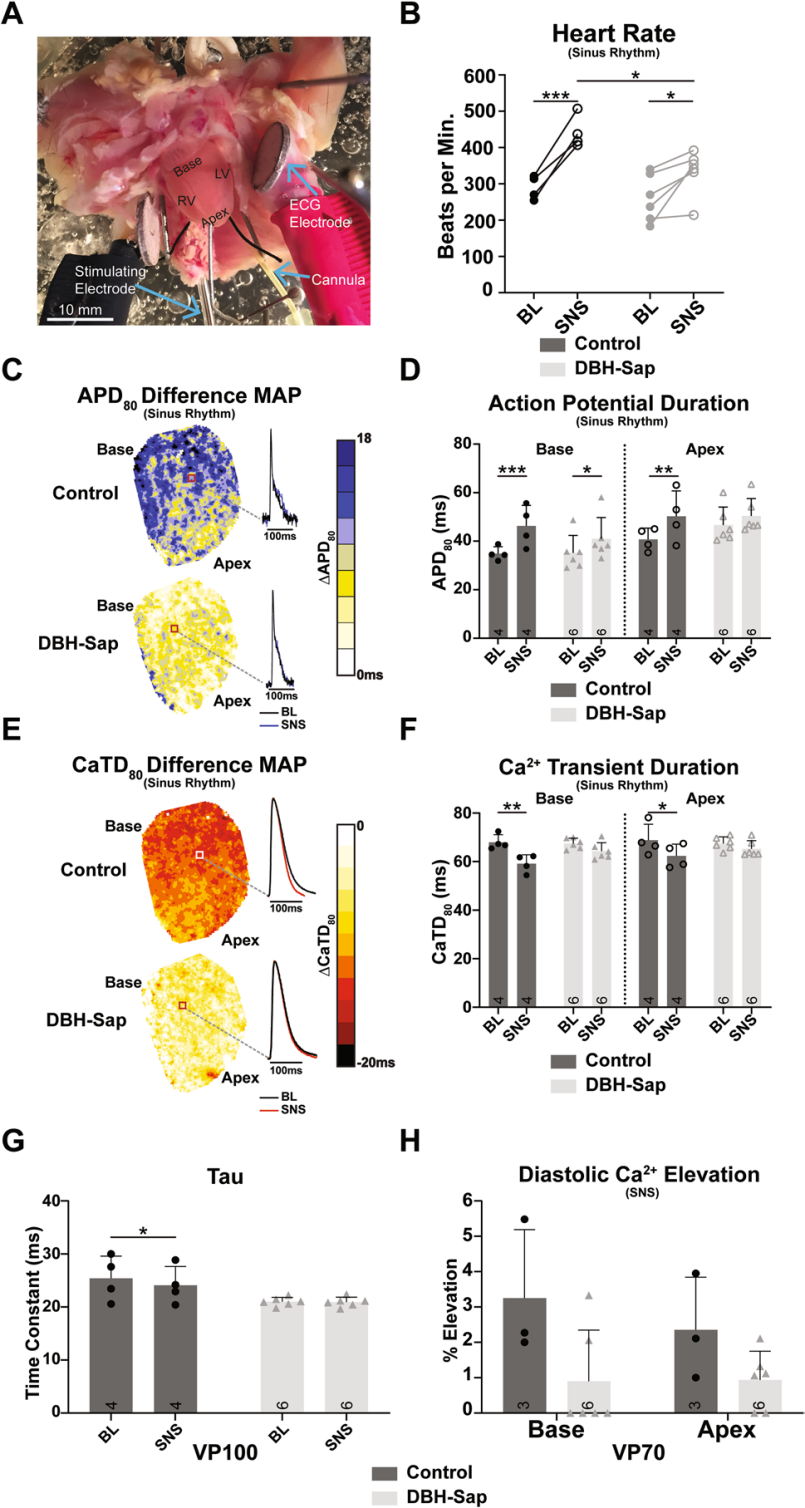
Electrophysiological responses to sympathetic nerve stimulation (SNS) during sinus rhythm and with pacing. (**A**) Photograph of the mouse innervated heart preparation. (**B**) Heart rate in control and DBH-Sap groups at baseline (BL) and with SNS. (**C**) Example difference maps (ΔAPD_80_) demonstrating APD prolongation with SNS compared to BL. (**D**) Mean APD_80_ from base and apex regions at BL and with SNS in both groups. (**E**) Example difference maps (ΔCaTD_80_) demonstrating CaTD shortening with SNS compared to BL. (**F**) Mean CaTD_80_ from base and apex regions at BL and with SNS in both groups. For panels (**B**–**F**) hearts are in sinus rhythm with and without SNS. (**G**) Time constant of Ca^2+^ decay (*tau)* of the whole heart at BL and with SNS in both groups at a pacing cycle length of 100 ms. (**H**) Mean diastolic Ca^2+^ elevation at base and apex regions of both groups following a 70 ms pacing train with SNS. Data are mean ± SD; analyzed with GraphPad Prism 8.3 (GraphPad Software, San Diego, CA, USA); control: n = 3–4; DBH-Sap: n = 6; *p < 0.05, **p < 0.01, ***p < 0.001.

Sympathetic nerve stimulation (SNS) was performed in innervated hearts to assess electrophysiological and Ca^2+^ handling responses to nerve-released norepinephrine (NE; Fig. 6A). Resting heart rates (HRs) were not different between groups, and HR significantly increased in both groups with SNS (Fig. 6B); however, control hearts had faster maximal HRs with SNS compared to DBH-Sap hearts (Control SNS: 442.0 ± 45.1 BPM vs. DBH-Sap: 333.2 ± 62.0 BPM, p < 0.05). We previously showed that SNS in the normal mouse heart leads to APD prolongation and CaTD shortening, while HR is increased^28^. Control hearts responded as expected, with APD prolongation and CaTD shortening at both the base and apex during SNS (Fig. 6C–F). In the DBH-Sap hearts, APD prolongation was observed at the base, but not apex (Fig. 6C,D) and CaTD did not change in either region with SNS (Fig. 6E,F). Likewise, the time constant of Ca^2+^ decay (*tau*) was not changed with SNS in the DBH-Sap group (Fig. 6G). To further evaluate Ca^2+^ handling dynamics with SNS, we also assessed diastolic Ca^2+^ elevation during SNS (PCL = 70 ms followed by 60-90 sec pause). SNS did not significantly alter diastolic Ca^2+^ elevation in either group (Fig. 6H).

## Discussion

Regional loss of cardiac sympathetic nerve fibers following MI has emerged as an indicator of ventricular arrhythmias and sudden cardiac death^12–16^. Yet, it remains difficult to assess which aspects of post-MI electrophysiological remodeling are due to ischemic damage versus nerve loss. The objective of this study was to determine the electrophysiological consequences of regional sympathetic hypo-innervation, similar to that occurring post-MI, but without the ischemia-induced myocardial damage. To our knowledge, this study is the first to utilize a targeted toxin to regionally lesion sub-epicardial cardiac sympathetic nerve fibers. By stimulating β-ARs with both a circulating agonist (ISO) and with physiological SNS, we investigated the effects of humoral and neuronal catecholamines, respectively, on AP and Ca^2+^ handling dynamics. In this study, we showed that (1) epicardial application of anti-DBH-Sap produces regional cardiac hypo-innervation (approximately 50% decrease in epicardial sympathetic fibers); (2) hypo-innervated hearts have super-sensitive responses to circulating ISO, including significant APD heterogeneity and regional diastolic Ca^2+^ elevation; and (3) hypo-innervated hearts showed muted HR, APD, and Ca^2+^ handling responses to physiological SNS.

Baseline AP dynamics between control and DBH-Sap hearts were similar. Following ISO, however, significant regional (primarily LV) APD shortening occurred in the DBH-Sap hearts but not control hearts (Fig. 3). This resulted in a significant increase in APD dispersion in the DBH-Sap hearts with ISO (Fig. 3F). These results are remarkably similar to our previous findings in a mouse model of reperfused MI, where we found dramatic APD shortening and increased APD dispersion in response to ISO in post-MI hearts with regional sympathetic nerve loss^11^. Furthermore, regional hyper-responsiveness to β-AR agonist application has also been observed in the clinical setting where post-infarct patients have significant changes in activation recovery intervals between the injury site and normal tissue, also resulting in increased dispersion^29^. Although we did not observe any reentrant arrhythmias in response to the pacing protocols performed in this study, increased APD dispersion within the heart is known to provide the substrate for unidirectional conduction block and reentry. We previously showed that reinnervation of the MI can reduce APD dispersion to nearly control levels, indicating that the scar itself may play a somewhat lesser role in contributing to APD heterogeneity (especially in response to circulating catecholamines) compared to the loss of sympathetic innervation^11,17^.

Altered Ca^2+^ handling is a hallmark of post-MI remodeling, which is often associated with abnormal sarcoplasmic reticulum (SR) Ca^2+^ release and reuptake^30,31^. We did not observe differences in the CaTD or *tau* (either at baseline or in response to ISO) between control and DBH-Sap hearts (Fig. 4). This may indicate that hypo-innervation alone is not sufficient to impair SR Ca^2+^ reuptake via the SERCA pump. Additionally, because the normal mouse heart relies heavily on SERCA for removal of cytosolic Ca^2+^ during relaxation (approx. 90% of Ca^2+^ is re-sequestered into the SR via SERCA and only 10% is extruded via the Na-Ca^2+^ exchanger)^32^, it is possible that with ISO stimulation, SERCA is maximally activated in both groups, and therefore super-sensitive responses are not observed.

Despite the fact that there were no differences in Ca^2+^ reuptake, β-AR stimulation with ISO resulted in significant diastolic Ca^2+^ elevation at the apex of DBH-Sap hearts, but not control after rapid ventricular pacing (Fig. 5A–D). During sympathetic stimulation, more Ca^2+^ is available in the cytosol due to increased SR Ca^2+^ load and a greater RyR open probability, resulting in greater cardiac contractility^33–35^. However, it appears as if the hypo-innervated hearts have increased RyR sensitivity and are more likely to open during diastole upon ISO stimulation. Importantly, though, this diastolic Ca^2+^ elevation was not large enough to trigger premature ventricular complexes (PVCs), suggesting that hypo-innervation may sensitize RyRs to circulating catecholamines, but additional injury mechanisms (e.g., fibrosis, cell–cell uncoupling) may be required for PVC generation^36^.

Consistent with this result, when recovery of Ca^2+^ release was assessed with an S1–S2 protocol, the apical region of DBH-Sap hearts showed accelerated recovery (Fig. 5F), again suggesting enhanced RyR sensitivity or decreased RyR refractoriness. We previously showed that accelerated recovery of Ca^2+^ release tends to decrease the formation of arrhythmogenic Ca^2+^ alternans^27^. Therefore, we hypothesized that the apical region of hypo-innervated hearts may be protected from Ca^2+^ alternans. Although there was a slight trend for decreased alternans at slower PCLs (Fig. 5H), this was not significant. Furthermore, at the fastest PCL (60 ms), DBH-Sap hearts had significantly *increased* Ca^2+^ alternans magnitude at both the base and the apex (Fig. 5G,H). This result is somewhat unexpected, considering that Ca^2+^ reuptake is not different between groups, and that Ca^2+^ release parameters would suggest *decreased* alternans. However, we previously observed significantly increased Ca^2+^ alternans magnitude in hypo-innervated post-MI hearts^11^. Therefore, the mechanisms contributing to hypo-innervation associated Ca^2+^ alternans remains an important area for future study.

In contrast to the humoral responses (i.e., circulating ISO), hypo-innervated hearts showed diminished ventricular responses to SNS, despite significant HR elevation, which indicates that atrial nerves are largely intact (Fig. 6B). We have previously shown that SNS in the rabbit heart produces shortening of APD and CaTD, while the mouse heart shows APD prolongation and CaTD shortening^37^. In the present study, we again show that nerve-released NE prolongs APD and shortens CaTD in control mouse hearts, but that this response to SNS is significantly diminished and does not occur in most regions of DBH-Sap hearts (Fig. 6D,F,G). Similarly, DBH-Sap hearts did not show any increases in diastolic Ca^2+^ elevation with SNS (Fig. 6H). These results are consistent with previous findings that indicate that β-AR activation only occurs at sites where the sympathetic nerve terminals and the corresponding cardiomyocyte are in direct contact. At these small junctional spaces, a relatively small quantity of NE produces a high local NE concentration to directly and efficiently stimulate β-ARs^38^. Thus, in hypo-innervated regions, we observed almost no changes in APD and CaTD nor diastolic Ca^2+^ elevation, because nerve loss leads to reduced and locally restricted NE release.

Catecholaminergic supersensitivity is a key feature of post-MI nerve loss, but the root cause within the β-AR pathway is not well understood. Zipes et al., have previously demonstrated that supersensitivity from sympathetic hypo-innervation in the canine model is not a result of a change in β-AR or α-subunit of G_s_ density or affinity, but may be due to changes in transduction further down the adrenergic signaling pathway^21,39^. Other studies have shown that a decrease in the β-AR regulatory protein, G-protein receptor kinase 2 (GRK2) may also play a role, since loss of GRK2 removes the negative feedback on β-AR activation^40^. Here, we have focused our assessments on functional outcomes, and discerning the cellular signaling involved in this model will be an important next step.

Cardiac sympathetic nerve loss also occurs in settings other than MI, including neurodegenerative diseases such as diabetic neuropathy^41^ and Parkinson’s disease^42^, as well as physiological aging^43^. Diabetic autonomic neuropathy (DAN) has a significant negative impact on the lives of diabetic patients, but is one of the least understood aspects of the disease. Cardiac consequences of DAN manifest as damage to the nerve fibers that innervate the heart and blood vessels, resulting in abnormalities in HR dynamics, increased risk of arrhythmias, and potential for SCD^41,44^. Likewise, clinical evidence suggests that patients with Parkinson’s disease may have supra-physiologic cardiovascular responses to adrenergic stimulation^45,46^, but detailed investigations of cardiac electrophysiological responses in these patients have not been performed. We previously showed that aged hearts have significantly decreased ventricular sympathetic nerve density, ventricular NE content, and reduced responsiveness to SNS^47^. Interestingly, this age-related neuro-degeneration did not create β-AR supersensitivity within the heart. Therefore, the underlying remodeling processes that produce β-AR supersensitivity in some pathological conditions but not others, remains an important area for future study.

## Conclusions

Heterogenous sympathetic stimulation coupled with the substrate of post-MI electrophysiological and structural remodeling is a highly arrhythmogenic combination, yet the impact of nerve loss versus myocardial remodeling is not well understood. In this study, we have replicated the catecholaminergic supersensitivity exhibited post-MI, but without ischemic myocardial damage. Regional sympathetic hypo-innervation produced supersensitivity to circulating catecholamines, while at the same time, reducing responsiveness to neuronal stimulation. The results of this study demonstrate the arrhythmogenic potential of sympathetic nerve heterogeneity and loss, even in the absence of the other confounding factors associated with post-MI remodeling.

### Limitations

In this study we utilized only male mice, but data indicate sex differences in age and rate of MI occurrence^3,48^, as well as in cardiac sympathetic innervation^49^. Thus, understanding the potential sex differences in response to regional hypo-innervation is an important area for future studies. In the present study, there was a relatively small sample size (n = 3–5) for some parameters due to variations in animal survival or in the signal-to-noise ratio of collected optical data; thus, conclusions must be interpreted accordingly. While α_1_-ARs are found in heart and may impact electrophysiological properties^50^, we only investigated β-AR sensitivity in the present study. α_1_-ARs may also have altered responsiveness following hypo-innervation and assessments of α_1_-AR sensitivity may provide additional insight into arrhythmia mechanisms following hypo-innervation. Likewise, neither α- or β-AR receptor density was quantified in the present study due to difficulties in precise dissection of hypo-innervated tissue without prior nerve labeling. Measurement of receptor density is therefore a key next step to further delineate underlying mechanisms.

## Materials and methods

### Ethical approval

All procedures involving animals were approved by the Animal Care and Use Committee of the University of California, Davis (protocol #20543), and complied with the Guide for the Care and Use of Laboratory Animals published by the National Institutes of Health. Male C57BL/6J mice (The Jackson Laboratory) were housed on a 12 h light/dark cycle with free access to standard chow and water in cages with no more than 5 mice per cage. UC Davis Teaching, Research, and Animal Care Services (TRACS) husbandry unit maintains the sanitation, equipment sterilization, chow and water replenishment, and room conditions and temperature (20–22 °C) of housing facilities. Post-surgical mice were singly housed. Mice were studied at 12–20 weeks of age, and randomly assigned to either DBH-Sap (n = 18) or control (n = 14) groups. Unequal n-numbers for optical mapping groups are a result of varying experimental success rates after initial randomization of animals to groups, or a result of insufficient optical signal-to-noise ratios for some signals or pacing protocols.

### Survival surgery

Mice were anesthetized with 2–3% inhaled isoflurane, intubated, and ventilated (MiniVent Model 845, Harvard Apparatus, Holliston, MA) with a 150 µL tidal volume at 120 breaths/min of isoflurane (1–2%) and oxygen. A lead I electrocardiogram (ECG) was continuously monitored. Thoracotomy at the fourth intercostal space allowed for the visualization of the anterior epicardial surface of the heart. The pericardium was gently opened. Either 5µL of 40 ng/µL of room temperature anti-dopamine beta-hydroxylase antibody conjugated to saporin (anti-DBH-SAP) or untargeted anti-immunoglobulin G antibody conjugated to saporin (anti-IgG-SAP—also termed ‘control’) (Advanced Targeting Systems, San Diego, CA) diluted in sterile saline was applied three times directly to the exposed apical/anterior surface of the heart. An interval of 15–30 s was allowed between each application, and 5 min after the third application before suturing the chest closed. As the mouse was graded off of the ventilator, 150 µL sterile saline (I.P.) and buprenex (0.1 mg/kg S.C.) were administered. Buprenex (0.1 mg/kg S.C.) and 100 µL saline were administered every 12 h for the next 48 h.

### Heart perfusion

On day 5 post-surgery, mice were administered 100 IU (I.P.) of heparin and anesthetized with pentobarbital sodium (150 mg/kg, I.P.). For traditional Langendorff perfusion, hearts were excised via a mid-sternal incision, submerged in cold cardioplegia solution (composition in mmol/L: NaCl: 110, CaCl_2_: 1.2, KCl: 16, MgCl_2_: 16, and NaHCO_3_: 10), and cannulated at the ascending aorta. For the innervated heart preparation, the thoracic cavity was isolated with spinal column (T1–T12 vertebrae) and sympathetic nerve fibers preserved. Following isolation, the innervated preparations were submerged in cold cardioplegia solution and then cannulated via the descending aorta. All hearts were retrograde perfused through the aorta with Tyrode’s solution at 37 ± 0.5 °C (composition in mmol/L: NaCl: 128.2, CaCl_2_: 1.3, KCl: 4.7, MgCl_2_: 1.05, NaH_2_PO_4_: 1.19, NaHCO_3_: 20 and glucose: 11.1). Perfusion flow rate was adjusted (2.5–4 mL/min) to maintain a perfusion pressure of 60–80 mmHg for the Langendorff preparation and 80–100 mmHg (7–10 mL/min) for the innervated preparation. Blebbistatin (10–20 µM, Tocris Bioscience, Ellisville, MO), an excitation–contraction uncoupler, was added to the perfusate to reduce motion artifacts during optical recordings^37^. The hearts were superfused in a dish with warm Tyrode’s, and three Ag/AgCl needle electrodes were positioned (two on either side of the heart and one reference in the dish) for continuous lead I ECG recording. A bipolar pacing electrode was placed on the LV (base for the Langendorff hearts and apex for the innervated hearts) for epicardial pacing.

### Dual optical mapping

Hearts were perfused with membrane voltage-sensitive (V_m_; RH237, 8-15µL of 1 mg/mL in DMSO; Biotium, Hayward, CA) and calcium-sensitive (Ca^2+^; Rhod2-AM, 20µL for Langendorff heart and 50 µL for innervated heart of 1 mg/mL in DMSO + 10% Pluronic acid; Biotium, Hayward, CA) indicators through coronary perfusion. The anterior epicardial surface was illuminated with LED light sources centered at 530 nm and bandpass-filtered from 511–551 nm (LEX-2, SciMedia, Costa Mesa, CA). Fluorescence emission was collected through a THT-macroscope (SciMedia, Costa Mesa, CA) and divided by a dichroic mirror at 630 nm into two paths (Omega, Brattleboro, VT). One light path longpass-filtered the RH237 signal at 700 nm and the other bandpass-filtered the Rhod2-AM signal at 574–606 nm^37^. Individual fluorescent signals were captured using two CMOS cameras (MiCam Ultima-L, SciMedia, Costa Mesa, CA) with a field of view of 10 × 10 mm at a sampling rate of 1 kHz. The effective spatial resolution was 100 μM/pixel.

### Experimental protocol

For Langendorff-perfused hearts, a pacing protocol of continuous ventricular pacing (pacing cycle lengths [PCLs] at regular intervals from 150 to 60 ms or until loss of capture), S1-S2 pacing (S1 = 150 ms; S2 = 130 ms to 60 ms), and 60–90 s of rapid ventricular pacing (70 ms PCL) was implemented to establish baseline parameters. Following the baseline assessment, hearts were challenged with isoproterenol (ISO, 300 nm–1 μm) to assess responsiveness to adrenergic stimulation.

For the innervated hearts, a catheter electrode (2F octapolar-pacing catheter; 0.2 mm electrode, 0.5 mm spacing; CIBer Mouse-EP Catheter; NuMed Inc, Hopkinton, NY) was inserted into the spinal column up to the T1-T3 vertebrae for activation of the sympathetic nerves projecting to the heart. Sympathetic nerve stimulation (SNS) frequency thresholds were determined by modulating the frequency of stimulation until the lowest frequency to initiate a heart rate (HR) increase was determined. Threshold testing began by first stimulating at 2 Hz, 7.5 V for 10 s and increasing frequency by 0.5 Hz for 10 s at constant voltage until a HR response of at least a 5% increase was observed. A working SNS frequency of 5 Hz above the threshold frequency was utilized for all subsequent protocols. The same pacing protocols as the Langendorff hearts were performed with and without SNS.

### Immunohistochemistry

#### Whole-heart immunohistochemistry

Sympathetic nerve fibers were labeled in a subset of intact hearts (n = 6) as previously described^51^. Briefly, whole hearts were fixed in formalin for 24 h at 4 °C. Hearts were then bleached in Dent’s bleach (4:1:1 methanol:DMSO:hydrogen peroxide) for 1 week at 4 °C with continuous agitation on a rocker. Hearts were then rehydrated in a series of descending methanol (MeOH)/PBS dilutions at 100%, 75%, 50%, and 25% MeOH:PBS for 1 h each. Hearts were permeabilized with 1% Triton-X 100/PBS (PBS-T) 3 × for 1 h each at room temperature and placed in 5% BSA/0.2% Sodium Azide (NaN)/PBS-T blocking solution overnight. The next morning, hearts were incubated with primary rabbit anti-tyrosine hydroxylase (TH) antibody (EMD Millipore) at 1:1000 BSA/NaN/PBS-T for 1 week at 4° C with continuous agitation. After 1 week, hearts were washed 3 × in PBS-T for 15 min each before being placed in biotin-conjugated donkey anti-rabbit IgG secondary antibody (EMD Millipore) at 1:200 BSA/NaN/PBS-T for 4 days at 4 °C with continuous agitation. Hearts were then washed 3 × for 15 min in PBS-T before being incubated in Vectastain ABC kit (per manufacturer’s instructions; Vector Laboratories) for 3 h at room temperature. Then the hearts were pre-incubated in Clear Stable DAB/Plus (Abcam) substrate buffer for 1 h at room temperature. Finally, Stable DAB was added dropwise. Hearts were stored in milli-Q water and imaged under a dissecting microscope.

#### Immunohistochemistry of frozen short-axis sections

Following optical mapping, a subset of hearts (control n = 3; DBH-Sap n = 3) were fixed in 4% paraformaldehyde for 1 h, rinsed with PBS, and placed in 30% sucrose/PBS overnight. Then 2 mm short-axis sections of the heart were cut and embedded in optimal cutting temperature (OCT) medium, frozen over dry ice, and stored at − 80 °C. Hearts were later sectioned into 10 µm short-axis sections, thaw-mounted onto positively charged slides, and stored at − 80 °C. TH labeling was performed as previously described^17^. After rehydration with PBS, slides were briefly incubated in sodium borohydride (10 mg/mL; 3 × 10 min) to reduce background auto-fluorescence. Slides were then blocked in 2% bovine serum albumin (BSA, Sigma) and 0.3% Triton X-100 (Sigma) in PBS (BSA/PBS-T) for 1 h. After a PBS wash, slides were incubated with primary rabbit anti-TH antibody (EMD Millipore) at 1:300 BSA/PBS-T overnight. Slides were then rinsed with PBS and incubated in Alexa Fluor 488 rabbit anti-Mouse (1:500 BSA/PBS-T, Invitrogen) secondary antibody for 1.5 h. After another PBS rinse, slides were dipped briefly in MilliQ water before being placed in a 10 mM copper sulfate/50 mM ammonium acetate solution for 30 min to further reduce background auto-fluorescence. Lastly, slides were dipped in MilliQ water and coverslipped with a 1:1 glycerol/PBS solution and immediately imaged. All slides were imaged on an upright Nikon Eclipse Ni microscope at 10 × magnification with a FITC filter (Ex/Em: 495/519 nm).

A total of three sections (base, mid, and apex regions) per heart were imaged in each group to determine percent TH + positive tissue area. The threshold tool of the Nikon NIS-Elements software was used to identify sympathetic nerve fibers. Two threshold criteria were established, one to identify TH-positive tissue and another to identify all tissue in the selection. Thus, percent TH-positive nerve area versus tissue area was calculated^52^. Images were de-identified and thresholded independently by two users.

#### Masson’s trichrome staining

Short-axis sections (control n = 3; DBH-Sap n = 3) preserved on slides at − 80 °C were utilized for Masson’s trichrome staining according to the manufacturer’s instructions (Trichrome Stain [Masson] Kit, Millipore-Sigma).

### Data analysis

Two analysis programs (*Optiq*, Cairn Research Ltd, UK & *Electromap*^53^) were utilized to analyze and interpret optical mapping data. A spatial Gaussian filter (3 × 3 pixels) was used to post-process all fluorescent signals. Masks of the epicardial surface were utilized for whole-heart analysis, as well as 10 × 10 pixel regions from the LV apex and base for region-dependent analysis. Both action potential (AP) and calcium transient (CaT) activation characteristics were measured as the time at 50% of the maximal amplitude from baseline, while repolarization times were calculated at 80% return to baseline. AP and CaT durations at 80% repolarization (APD_80_; CaTD_80_) were calculated as repolarization time minus activation time. APD dispersion (APDD) was calculated as the inner 5th to 95th percentile of APDs in the field of view divided by the median APD. Average conduction velocity (CV) was calculated along a gradient vector of the surface from base to apex using a polynomial fitting algorithm to measure activation times as described by O’Shea et al^53^. Diastolic Ca^2+^ elevation was measured as the percent increase in diastolic Ca^2+^ relative to the last paced beat in a rapid pacing train. CaT decay (tau) was calculated as the time constant of decay of a single exponential fit to the recovery portion of the CaT (from 30–90% of baseline). S2/S1 ratio of CaT amplitudes were calculated to assess recovery of relative Ca^2+^ release in response to premature stimuli. CaT alternans analysis was conducted using 6–8 consecutive beats at each PCL of continuous pacing and quantified via spectral methods as previously described^27^.

### Statistics

All data are presented as mean ± standard deviation (SD). All data were analyzed by two-way ANOVA (repeated measures where appropriate) with Sidak’s multiple comparisons post-testing as recommended by GraphPad Prism 8.3 (GraphPad Software, San Diego, CA, USA). P < 0.05 was considered statistically significant.

## Data Availability

The data generated for the current study are available from the corresponding author on reasonable request.
